# Association between cumulative smoking exposure and cognitive decline in non-demented older adults: NEDICES study

**DOI:** 10.1038/s41598-023-32663-9

**Published:** 2023-04-08

**Authors:** Julián Benito-León, Ritwik Ghosh, José Lapeña-Motilva, Cristina Martín-Arriscado, Félix Bermejo-Pareja

**Affiliations:** 1 Department of Neurology, University Hospital “12 de Octubre”, Avda. De Córdoba km. 5,400, 28041 Madrid, Spain; 2 Centro de Investigación Biomédica en Red sobre Enfermedades Neurodegenerativas (CIBERNED), Madrid, Spain; 3 Department of Medicine, Complutense University, Madrid, Spain; 4 Research Institute (imas12), University Hospital “12 de Octubre”, Madrid, Spain; 5 Department of General Medicine, Burdwan Medical College, Burdwan, West Bengal India

**Keywords:** Cognitive ageing, Neuroscience, Neurology

## Abstract

Whether cumulative smoking exposure is associated with cognitive decline among older adults remains unresolved. To address this question, we used data from the Neurological Disorders in Central Spain (NEDICES) cohort study, in which 2624 older adults were evaluated at two-time points separated by three years. A 37-item version of the Mini-Mental State Examination (MMSE-37) was administered at two visits to assess cognitive change. Regarding smoking exposure, we calculated an individual baseline score based on pack-years (i.e., packs of cigarettes smoked per day multiplied by years of smoking) in current and former smokers. Thus, smoking exposure was categorized into tertiles (low: < 19.0, medium: 19.0–47.0, and high: > 47.0). We used multivariable generalized estimating equation models to assess associations between pack-years and smoking status with 37-MMSE total score change from baseline to follow-up. The MMSE-37 total score had a decline of 1.05 points (confidence interval [CI] 95% 0.62 to 1.48) in the lower tertile of pack-years, 1.16 (CI 95% 0.70 to 1.62) in the middle tertile and 1.17 (CI 95% 0.70 to 1.65) in the higher tertile compared to never smokers, after adjusting for several demographic and clinical variables. The same occurred with smoking status, i.e., a decline of 1.33 (CI 95% 0.87 to 1.79) in current smokers and 1.01 (CI 95% 0.63 to 1.40) in former smokers. Our study provides evidence of the cumulative effect of smoking on cognition in older adults. Using a prospective population-based design, we demonstrated that cumulative smoking exposure was associated with cognitive decline in non-demented older adults. More population-based evidence is required to elucidate this association in older adults without dementia.

## Introduction

Dementia is currently the seventh leading cause of death and remains one of the major causes of long-term disability and dependency worldwide^1^. Despite continuous scientific research, therapeutic approaches to prevent deterioration have limited efficacy, whereas the interest in novel risk and preventive factors is increasing^2^. In this context, vascular diseases have been among the most established risk factors for dementia in older adults^3^. Smoking exposure itself is a potent risk factor, even in low doses, for vascular diseases, including stroke^4–6^. These facts are consistent with observational studies revealing a consistent association between current smoking status and dementia; however, the relationship between smoking exposure and cognitive decline is not consistent across scientific literature^2,7–9^.

Prospective population-based studies are preferential to investigate risk factors for cognitive decline^10,11^. However, the few population-based studies that have addressed the association between smoking and cognitive decline have shown discrepant results^7,9,12,13^. Controlling unmeasured confounders, such as sedentary lifestyle^14^ or depressive symptoms^15^, may shed light on this heterogeneity.

Hence, whether smoking exposure is associated with cognitive decline among older adults remains unresolved. To address this question, we used data from the Neurological Disorders in Central Spain (NEDICES) cohort study, in which 2624 (≥ 65 years) participants were prospectively evaluated at two time points separated by 3 years^16–18^. We tested the hypothesis that the higher the exposure to smoking among non-demented older adults, the higher the cognitive decline.

## Methods

### Ethical aspects

All the participants included in the study gave their written informed consent after a full explanation of the procedure. The study, which was conducted per the principles of the Helsinki declaration of 1975, was approved by the ethical standards committee on human experimentation at the University Hospitals "12 de Octubre" and "La Princesa" in Madrid. Written (signed) informed consent was obtained from all enrollees.

### Study population

Data for these analyses were derived from the NEDICES study, a longitudinal, population-based survey of the prevalence, incidence, and determinants of primary age-associated conditions in older people^16–18^. Detailed accounts of the study population and sampling methods have been published elsewhere^16–18^.

The survey area consisted of three communities: (1) Las Margaritas (approximately 14,800 inhabitants), a working-class neighborhood in Getafe (Greater Madrid); (2) Lista (approximately 150,000 inhabitants), a professional-class neighborhood in Central Madrid; and (3) Arévalo (approximately 9000 inhabitants), the agricultural zone of Arévalo County (125 km northwest of Madrid)^16–18^. Up-to-date lists of residents were generated from population registers. The eligibility was restricted to residents aged 65 years or older living there on December 31, 1993, or during six or more months of 1993^16–18^. Eligible persons who had moved away from the survey area were not traced. In Margaritas and Arévalo, every eligible was screened, but proportionate stratified random sampling was used for screening in Lista due to a large number of older adult residents^16–18^.

### Study evaluation

Briefly, at their baseline assessment (1994–1995), 5278 older people were interviewed using a 500-item screening questionnaire to collect data on demographics, medications, current medical conditions, smoking (current, former, and never), and drinker (current/at least once per week, former, and never)^16–18^. A comorbidity index was calculated based on the following conditions: atrial fibrillation, cancer, chronic obstructive pulmonary disease, depression, dementia, diabetes, epilepsy (treated), heart failure, myocardial infarction, psychiatric disorders, renal disease, and stroke^19^. In addition, participants were asked to rate their current health using a 5-point scale using the question, "In general terms, how would you describe your health: much better, better, similar, worse, or much worse?"^20,21^. Response options were collapsed into three categories (much better and better, similar, and worse/much worse)^21,22^. A Spanish adaptation of the Pfeffer Functional Activities Questionnaire (FAQ) was administered to participants (a higher score means a lower cognitive and motor functional activity function)^23^.

To assess health habits, each participant was asked to indicate their "total hours of actual sleep in 24 h"^24,25^. Participants indicated their usual daily sleep duration (i.e., total) as the sum of nighttime sleep and daytime napping^24,25^. Besides, a modified version of the Rosow–Breslau questionnaire was applied to categorize physical activity into active versus sedentary groups^14,26^.

The same methods were used during the second (i.e., follow-up) evaluation (1997–1998).

### Cognitive assessment

A 37-item version of the Mini-Mental State Examination (MMSE-37) was administered at both the baseline assessment (1994–1995) and the follow-up assessment (1997–1998). This was a Spanish adaptation of the standard MMSE^27,28^, which included all the standard MMSE items and three additional tasks: (1) an attention task, i.e., "say 1, 3, 5, 7, 9 backward", (2) a visual order, i.e., a man raising his arms, and (3) a simple construction task, i.e., copying two overlapping circles^27,28^.

The diagnosis of dementia was assigned by consensus of expert neurologists using the Diagnostic and Statistical Manual of Mental Disorders (DSM)—IV criteria^29^.

### Final selection of participants

Of the 5278 participants evaluated at baseline, we excluded 467 participants with a diagnosis of dementia: 306 with dementia at baseline evaluation (1994–1995) (i.e., prevalent cases)^30^ and 161 who developed dementia at follow-up (1997–1998) (i.e., incident cases)^3^. Further, 2187 participants were excluded due to different reasons: a) Follow-up assessment was declined or incomplete (i.e., demised or unreachable; N = 1246), MMSE-37 (N = 938) or smoking status (N = 3) was not available. The eligible sample (N = 2624) was similar to the base sample (N = 5278) in terms of sex (1485 [56.6%] vs. 3040 [57.6%] women, chi-square = 0.72, p = 0.395), but they were more educated (268 [10.1%] vs. 711 [13.6%] were illiterate, chi-square = 18.58, p < 0.001) and younger (72.7 ± 5.9 vs. 74.3 ± 7.0 years, t = 11.0, p < 0.001).

The final sample (N = 2624) was followed-up during a mean of 3.3 years (range 1.5–4.7 years) (Table 1).

**Table 1:** Baseline demographic and clinical characteristics of participants (N = 2624).

Age (years)	72.7 (71.0) ± 5.9
Sex (women)	1485 (56.6%)
Years of education	7.0 (7.0) ± 5.3
Sleep duration (hours per day)*	
≤ 5	284 (10.9%)
6–8	1288 (49.5%)
≥ 9	1032 (39.6%)
Comorbidity index	0.9 (0.0) ± 1.3
Body mass index*	27.8 (27.3) ± 5.5
Subjective wellbeing*	
Much better and better	1596 (61.0%)
Similar	757 (28.9%)
Worse and much worse	262 (10.0%)
Physical activity*	
Sedentary lifestyle	558 (22.7%)
Mild	712 (29.0%)
Moderate	550 (22.4%)
High	636 (25.9%)
Pfeffer Functional Activities Questionnaire total score*	1.2 (0.0) ± 3.1
Drinking status*	
Current drinkers	879 (35.6%)
Former drinkers	476 (19.3%)
Never-drinkers	1111 (45.0%)
Smoking status	
Current smokers	305 (11.6%)
Former smokers	727 (27.7%)
Never-smokers	1592 (60.7%)

Mean (median) ± standard deviation and frequency (%) are reported.

*Data on some participants were missing.

### Statistical analyses

Statistical analyses were performed in SPSS Version 24.0 (SPSS, Inc., Chicago, IL) and Stata InterCooled for Windows version 16 (StataCorp. 2019. Stata Statistical Software: Release 16. College Station, TX: StataCorp LLC). None of the continuous variables (age, years of education, alcohol consumption (years), comorbidity index, body mass index, FAQ score, and baseline and follow-up MMSE-37 total score) were normally distributed (Kolmogorov–Smirnov, p < 0.001), even after log-transformation. Therefore, baseline characteristics scores were compared using Kruskal–Wallis tests. The Chi-square test was applied when appropriate to determine associations between categorical variables.

Regarding smoking exposure, we calculated an individual baseline score based on pack-years (i.e., packs of cigarettes smoked per day multiplied by years of smoking) in current and former smokers. Thus, smoking exposure was categorized into tertiles (low < 19.0, medium 19.0–47.0, high > 47.0). Participants who never smoked were coded as zero pack-years, and they served as the reference group. Otherwise, participants were classified according to their smoking status into three groups: never-smokers, former, and current smokers.

We used multivariable generalized estimating equation models to assess associations between pack-years and smoking status with 37-MMSE total score change from baseline to follow-up. Age (years), sex, years of education, sleep duration (≤ 5, 6–8 ≥ 9 h), comorbidity index, untreated arterial hypertension, body mass index, subjective wellbeing (much better and better, similar, and worse and much worse), level of physical activity (sedentary, mild, moderate, and high), FAQ score, and alcohol status (current and former drinkers, and never-drinkers) were considered potential variables. In the multivariate model, we adjusted for those baseline variables with p-values lower than 20% associated with cumulative smoking exposure (Table 2).

**Table 2:** Participants' baseline demographic and clinical characteristics stratified by cumulative smoking exposure (N = 2624).

	Never smokers (N = 1592)	Lower tertile of pack-years (< 19) (N = 344)	Middle tertile of pack-years (19–47) (N = 345)	Higher tertile of pack-years (> 47.0) (N = 343)	*p-*value
Age (years)	73.0 (72.0) ± 6.0	72.9 (71.0) ± 6.0	71.3 (70.0) ± 5.3	72.3 (71.0) ± 5.5	< 0.001^a^
Sex (women)	1348 (84.7%)	85 (24.7%)	34 (9.9%)	18 (5.2%)	< 0.001^b^
Years of education	6.5 (7.0) ± 4.5	8.1 (8.0) ± 5.8	8.3 (8.0) ± 6.8	7.2 (7.0) ± 5.7	< 0.001^a^
Sleep duration (hours per day)*					< 0.001^b^
≤ 5	204 (12.9%)	25 (7.3%)	25 (7.3%)	30 (8.8%)	
6–8	781 (49.5%)	179 (52.0%)	183 (53.4%)	145 (42.6%)	
≥ 9	592 (37.5%)	140 (40.7%)	135 (39.4%)	165 (48.5%)	
Comorbidity index	0.8 (0.0) ± 1.2	0.9 (0.0) ± 1.2	1.0 (0.0) ± 1.3	1.2 (1.0) ± 1.4	< 0.001^a^
Body mass index*	28.3 (27.5) ± 6.4	26.9 (26.8) ± 3.8	27.3 (27.2) ± 4.2	27.0 (26.8) ± 3.6	< 0.001^a^
Subjective wellbeing*					< 0.001^b^
Much better and better	905 (57.0%)	232 (68.2%)	241 (70.0%)	218 (63.7%)	
Similar	495 (31.2%)	85 (25.0%)	79 (23.0%)	98 (28.7%)	
Worse and much worse	189 (11.9%)	23 (6.8%)	24 (7.0%)	26 (7.6%)	
Physical activity*					0.028^b^
Sedentary lifestyle	312 (21.1%)	76 (23.2%)	76 (23.2%)	94 (29.4%)	
Mild	429 (28.9%)	90 (27.5%)	99 (30.3%)	94 (29.4%)	
Moderate	326 (22.0%)	75 (22.9%)	78 (23.9%)	71 (22.2%)	
High	415 (28.0%)	86 (26.3%)	74 (22.6%)	61 (19.1%)	
Pfeffer Functional Activities Questionnaire total score*	1.5 (0.0) ± 3.3	0.8 (0.0) ± 2.3	0.9 (0.0) ± 2.4	1.0 (0.0) ± 2.8	< 0.001^a^
Alcohol status*					< 0.001^b^
Current drinker	307 (20.6%)	191 (58.8%)	190 (58.8%)	191 (58.2%)	
Former drinker	227 (15.2%)	78 (24.0%)	78 (24.1%)	93 (28.3%)	
Never-drinker	956 (64.2%)	56 (17.2%)	55 (17.0%)	44 (13.4%)	

^a^Kruskal–Wallis test; ^b^Chi-square test. *Data on some participants were missing. Mean (median) ± standard deviation and frequency (%) are reported. *MMSE-37* 37-item version of the Mini-Mental State Examination.

## Results

A description of the sample concerning cumulative smoking exposure is shown in Table 2. At baseline, those who never smoked were older, less educated, used to drink less, scored higher on the FAQ score, and rated their health as poorer. Moreover, they were women in a higher proportion, showing a higher body mass index and depressive symptoms. The participants within the highest tertile of pack years had more comorbidities, their lifestyle was more sedentary, and they slept more hours than never-smokers and those in the middle and lower tertiles.

Table 3 shows the generalized estimating equation models for the association between cumulative smoking exposure and smoking status with MMSE-37-change between the two visits (the baseline and the follow-up assessments). The MMSE-37 total score had a decline of 1.05 points (confidence interval [CI] 95% 0.62 to 1.48) in the lower tertile of pack-years (< 19.0), 1.16 (CI 95% 0.70 to 1.62) in the middle tertile (19.0–47.0) and 1.17 (CI 95% 0.70 to 1.65) in the higher tertile (> 47.0) compared to never smokers, after adjusting for age, sex, years of education, sleep duration (≤ 5, 6–8 ≥ 9 h), comorbidity index, body mass index, subjective wellbeing (much better and better, similar, and worse and much worse), level of physical activity (sedentary, mild, moderate, and high), FAQ score, and alcohol status (current and former drinkers, and never-drinkers) (Table 3). The same occurred with smoking status, i.e., the MMSE-37 total score had a decline of 1.33 (CI 95%  0.87 to 1.79) in current smokers and 1.01 (CI 95% 0.63 to 1.40) for former smokers to never-smokers (Table 3). In sum, both accumulative exposure to smoking and smoking status (current and former smokers) were associated with an MMSE-37 total score decline than never-smokers.

**Table 3:** Multivariable generalized estimating equation models to assess associations between pack-years and smoking status with 37-MMSE total score change from baseline to follow-up.

	Cumulative smoking exposure			Smoking status		
	Coefficient	95% confidence interval	*p*-value	Coefficient	95% confidence interval	*p*-value
Age (years)	− 0.14	− 0.16 to − 0.11	< 0.001	− 0.14	− 0.16 to − 0.11	< 0.001
Sex						
Women	− 0.90	− 1.30 to − 0.51	0.001	− 0.93	− 1.33 to 0.53	0.001
Men (ref.)						
Years of education	0.24	0.21 to 0.27	< 0.001	0.24	0.21 to 0.26	< 0.001
Sleep duration (h)						
≥ 9	− 0.09	− 0.43 to 0.24	0.582	− 0.10	− 0.44 to 0.24	0.128
≤ 5	− 0.38	− 0.88 to − 0.02	0.049	− 0.39	− 0.89 to 0.01	0.061
6–8 (ref.)						
Comorbidity index	0.12	0.01 to 0.22	0.035	0.12	0.00 to 0.23	0.032
Body mass index	− 0.03	− 0.05 to − 0.01	0.048	− 0.02	− 0.05 to 0.00	0.053
Subjective wellbeing						
Bad/very bad	− 1.55	− 2.02 to − 1.07	< 0.001	− 1.54	− 2.01 to − 1.07	< 0.001
Normal	− 0.73	− 1.03 to − 0.44	< 0.001	− 0.73	− 1.02 to − 0.44	< 0.001
Good/excellent (ref.)						
Physical activity						
High	0.89	0.40 to 1.38	0.001	0.91	0.42 to 1.40	0.001
Moderate	0.71	0.27 to 1.16	0.002	0.72	0.27 to 1.16	0.002
Mild	0.57	0.19 to 0.94	0.003	0.58	0.20 to 0.95	0.002
Sedentary lifestyle (ref)			−			
FAQ score	− 0.32	− 0.36 to − 0.27	< 0.001	− 0.30	− 0.35 to − 0.25	< 0.001
Alcohol status						
Current	− 0.69	− 1.02 to − 0.36	0.001	− 0.67	− 1.01 to − 0.34	0.001
Former drinker	− 0.42	− 0.79 to − 0.06	0.024	− 0.42	− 0.79 to − 0.06	0.023
Never-drinker (ref)						
Cumulative smoking exposure						
Higher tertile of pack-years (> 47)	− 1.17	− 1.65 to − 0.70	< 0.001	− 1.33 (current smoker)	− 1.79 to − 0.87	< 0.001
Middle tertile of pack-years (19–47)	− 1.16	− 1.62 to − 0.70	< 0.001	− 1.01 (former smoker)	− 1.40 to − 0.63	< 0.001
Lower tertile of pack-years (< 19)	− 1.05	− 1.48 to − 0.62	< 0.001			
Never smokers (ref.)						

## Discussion

This population-based study showed a dose-dependent relationship between cumulative smoking exposure and cognitive decline in non-demented older Spanish adults. Even light smokers had a cognitive decline. Moreover, we also identified an association with cognitive decline among current and former smokers. These findings highlight the importance of complete smoking abstinence for preventing cognitive decline.

The scientific literature concerning cognitive functioning and smoking in older adults is inconsistent^7,13,31^. A meta-analysis found that seven out of thirteen studies demonstrated a significant association with an increased risk of cognitive decline, whereas the remaining six revealed no significant link between dementia or cognitive decline and current smoking^7^. Moreover, the Rotterdam Study, which included middle-aged and older adults, demonstrated that current smoking was related to a decline in global cognition; there was no evidence for effect modification by APOE ɛ4 genotype on this relation^13^. By contrast, lifelong exposure to smoking was not associated with a cognitive decline in a prospective study of Alzheimer's disease outpatients from São Paulo, Brazil, independently of their APOE ɛ4 allele carrier status^31^.

Although the current findings suggest that smoking is associated with cognitive decline in older adults, the explanatory mechanisms remain unclear. Cerebral small vessel disease burden (i.e., the presence of white matter hyperintensities and lacunar infarcts) increases the risk of cognitive decline^32^, and it is well known that chronic smoking is associated with this cerebrovascular conditions^33–37^ Moreover, white matter degeneration is an early component of Alzheimer's disease and mild cognitive impairment^35^. Thus, smoking may contribute to white matter degeneration by inhibiting the expression of genes needed for myelin synthesis and maintenance, and, therefore, could be a key risk factor for several neurodegenerative diseases, including pre-AD stages^36^. A large cohort study of healthy older adults (N = 1111) demonstrated that current smokers had an increased annual hippocampus atrophy rate versus non or former smokers independently of lifetime tobacco consumption^37^. Taking these facts into account, chronic smoking exposure is associated with accelerated brain aging^38^.

This study has some limitations. Firstly, the final sample comprised 2624 due to the exclusions. Thus, many participants had died or were unreachable (N = 1246), and MMSE-37 data were missed (N = 938), commonly occurring in prospective population-based studies. Secondly, we only included Spanish participants; therefore, one could think that the results could not be extrapolated to other countries. However, our cohort comprised older people, with only 11.6% current smokers and 27.7% former smokers, prevalence rates similar in older people from other European countries, which permits extrapolating our results^39^. Thirdly, many countries still have negative social attitudes regarding women's smoking. As a result, female respondents in certain countries sometimes do not tell the truth about their smoking status. In large population-based studies, including NEDICES, it is difficult to measure bias or false labeling in female respondents' answers to smoking-related questions. However, the low prevalence of smoking among women participating in the NEDICES could be explained by the country's peculiarities. Historically, the uptake of smoking by Spanish women was delayed by social conditions that prevailed during the Spanish Civil War (1936–1939) and World War II (1939–1945) and by the General Francisco Franco dictatorship^40^. Most NEDICES cohort women spent their adolescence and early adulthood during those periods. These women were educated to be homemakers and to take care of their families^40^. The Franco regime encouraged traditional social roles for women, which prohibited cigarette usage, among other things. Indeed, smoking was not an option for that women generation^40^. In Spain, women's biggest cigarette smoking growth occurred during the transition from dictatorship to democracy (the 1970s)^40^. Fourthly, the MMSE-37 is a relatively abbreviated dementia screening tool; more formal neuropsychological testing is needed in future studies. However, even with this essential cognitive tool, we established differences among the established smoking groups. Fifthly, the presence of minor neurocognitive disorders was not discharged in the analyses. Finally, we did not adjust for other possible variables, such as blood glucose, lipid profiles, and APOE ɛ4 allele carrier status, which could influence the risk of cognitive decline. This study also had several strengths. Firstly, the study was population-based, allowing us to assess a broad group of unselected older adults. Secondly, the assessments were conducted prospectively in a standardized manner. Finally, we could adjust for the potential confounding effects of several essential factors.

This study provides evidence of the cumulative effect of smoking on cognition in older adults. Using a prospective population-based design, we demonstrated that cumulative smoking exposure was associated with cognitive decline in non-demented older adults. More population-based evidence is required to elucidate this association in older adults without dementia.

## Data Availability

Anonymized data will be shared by request from any qualified investigator and provided by Dr. Julián Benito-León (jbenitol67@gmail.com).

## References

[1] Cahill S (2020). WHO's global action plan on the public health response to dementia: Some challenges and opportunities. Aging Ment. Health.

[2] Livingston G (2017). Dementia prevention, intervention, and care. Lancet.

[3] Bermejo-Pareja F, Benito-León J, Vega S, Medrano MJ, Román GC (2008). Neurological Disorders in Central Spain (NEDICES) Study Group. Incidence and subtypes of dementia in three elderly populations of central Spain. J. Neurol. Sci..

[4] Wolf PA, D’Agostino RB, Belanger AJ, Kannel WB (1991). Probability of stroke: A risk profile from the Framingham Study. Stroke.

[5] Chambless LE, Heiss G, Shahar E, Earp MJ, Toole J (2004). Prediction of ischemic stroke risk in the Atherosclerosis Risk in Communities Study. Am. J. Epidemiol..

[6] Hackshaw A, Morris JK, Boniface S, Tang JL, Milenković D (2018). Low cigarette consumption and risk of coronary heart disease and stroke: Meta-analysis of 141 cohort studies in 55 study reports. BMJ.

[7] Peters, R. *et al.* Smoking, dementia and cognitive decline in the elderly, a systematic review. *BMC Geriatr.* **8** (2008). 10.1186/1471-2318-8-36 PMC2642819 19105840

[8] Anstey KJ, Sanden C, Salim A, O'Kearney R (2007). Smoking as a risk factor for dementia and cognitive decline: A meta-analysis of prospective studies. Am. J. Epidemiol..

[9] Deal JA (2020). Relationship of cigarette smoking and time of quitting with incident dementia and cognitive decline. J. Am. Geriatr. Soc..

[10] Benito-León J, Vega-Quiroga S, Villarejo-Galende A, Bermejo-Pareja F (2015). Hypercholesterolemia in elders is associated with slower cognitive decline: A prospective, population-based study (NEDICES. J. Neurol. Sci..

[11] Contador I, Bermejo-Pareja F, Pablos DL, Villarejo A, Benito-León J (2017). High education accelerates cognitive decline in dementia: A brief report from the population-based NEDICES cohort. Dement. Neuropsychol..

[12] Mons U, Schöttker B, Müller H, Kliegel M, Brenner H (2013). History of lifetime smoking, smoking cessation and cognitive function in the elderly population. Eur. J. Epidemiol..

[13] Wingbermühle R, Wen KX, Wolters FJ, Ikram MA, Smoking BD (2017). APOE genotype, and cognitive decline: The Rotterdam study. J. Alzheimers Dis..

[14] Llamas-Velasco S, Contador I, Villarejo-Galende A, Lora-Pablos D, Bermejo-Pareja F (2015). Physical activity as protective factor against dementia: A prospective population-based study (NEDICES). J. Int. Neuropsychol. Soc..

[15] Olazarán, J., Trincado, R. & Bermejo-Pareja, F. Cumulative effect of depression on dementia risk. *Int. J. Alzheimers Dis.* **2013**, (2013). 10.1155/2013/457175 PMC3789404 24159419

[16] Morales JM (2004). Methods and demographic findings of the baseline survey of the NEDICES cohort: A door-to-door survey of neurological disorders in three communities from Central Spain. Public Health.

[17] Bermejo-Pareja F (2008). La cohorte de ancianos NEDICES. Metodología y principales hallazgos neurológicos [The NEDICES cohort of the elderly. Methodology and main neurological findings]. Rev. Neurol..

[18] Vega S (2010). Several factors influenced attrition in a population-based elderly cohort: Neurological disorders in Central Spain Study. J. Clin. Epidemiol..

[19] Carey IM, Shah SM, Harris T, DeWilde S, Cook DG (2013). A new simple primary care morbidity score predicted mortality and better explains between practice variations than the Charlson index. J. Clin. Epidemiol..

[20] Fernández-Ruiz M (2013). The ability of self-rated health to predict mortality among community-dwelling elderly individuals differs according to the specific cause of death: Data from the NEDICES cohort. Gerontology.

[21] Benito-León J, Louis ED, Villarejo-Galende A, Labiano-Fontcuberta A, Bermejo-Pareja F (2015). Self-rated health and risk of incident essential tremor: A prospective, population-based study (NEDICES). Parkinson. Relat. Disord..

[22] Benito-León J, Romero JP, Louis ED, Bermejo-Pareja F (2014). Faster cognitive decline in elders without dementia and decreased risk of cancer mortality: NEDICES Study. Neurology.

[23] Louis ED, Benito-León J, Vega-Quiroga S, Bermejo-Pareja F (2010). Neurological Disorders in Central Spain (NEDICES) Study Group. Cognitive and motor functional activity in non-demented community-dwelling essential tremor cases. J. Neurol. Neurosurg. Psychiatry.

[24] Benito-León J, Louis ED, Bermejo-Pareja F (2013). Cognitive decline in short and long sleepers: A prospective population-based study (NEDICES. J. Psychiatr. Res..

[25] Benito-León J, Louis ED, Villarejo-Galende A, Romero JP, Bermejo-Pareja F (2014). Long sleep duration in elders without dementia increases risk of dementia mortality (NEDICES). Neurology.

[26] Bermejo-Pareja F (2022). The health status: The ignored risk factor in dementia incidence. NEDICES cohort. Aging Clin. Exp. Res..

[27] Serna A (2015). Accuracy of a brief neuropsychological battery for the diagnosis of dementia and mild cognitive impairment: An Analysis of the NEDICES cohort. J. Alzheimers Dis..

[28] Contador I (2016). The 37 item Version of the Mini-Mental State Examination: Normative data in a population-based cohort of older spanish adults (NEDICES). Arch. Clin. Neuropsychol..

[29] Diagnostic and statistical manual of mental disorders, 4th ed. xxvii, 886–xxvii, 886 (1994).

[30] Bermejo-Pareja F (2009). Consistency of clinical diagnosis of dementia in NEDICES: A population-based longitudinal study in Spain. J. Geriatr. Psychiatry Neurol..

[31] de Oliveira FF (2018). Lifetime risk factors for functional and cognitive outcomes in patients with Alzheimer's disease. JAD.

[32] Cerebral small vessel disease burden and longitudinal cognitive decline from age 73 to 82: The Lothian Birth Cohort 1936. *Transl. Psychiatry* **11**(1), 376 (2021). 10.1038/s41398-021-01495-4 PMC8257729 34226517

[33] Kim, S. H. *et al.* Age-dependent association between cigarette smoking on white matter hyperintensities. *Neurol. Sci.* **33**(1):45–51 (2012). 10.1007/s10072-011-0617-1 21562840

[34] Howard G (1998). Cigarette smoking and other risk factors for silent cerebral infarction in the general population. Stroke.

[35] Amlien IK, Fjell AM (2014). Diffusion tensor imaging of white matter degeneration in Alzheimer's disease and mild cognitive impairment. Neuroscience.

[36] Yu R (2016). Tobacco smoke-induced brain white matter myelin dysfunction: Potential co-factor role of smoking in neurodegeneration. J. Alzheimers Dis..

[37] Duriez, Q., Crivello, F. & Mazoyer, B. Sex-related and tissue-specific effects of tobacco smoking on brain atrophy: assessment in a large longitudinal cohort of healthy elderly. *Front Aging Neurosci* **6**, (2014). 10.3389/fnagi.2014.00299 PMC4217345 25404916

[38] Linli, Z., Feng, J., Zhao, W. & Guo, S. Associations between smoking and accelerated brain ageing. *Prog. Neuropsychopharmacol. Biol. Psychiatry*. **113** (2022). 10.1016/j.pnpbp.2021.110471 34740709

[39] Lugo A, La Vecchia C, Boccia S, Murisic B, Gallus S (2013). Patterns of smoking prevalence among the elderly in Europe. IJERPH.

[40] Enders, V. L. & Radcliff, P. B. *Constructing Spanish Womanhood: Female Identity in Modern Spain*. (State University of New York Press, 1998).

